# Efficacy and Safety of Wei Bi Mei, a Chinese Herb Compound, as an Alternative to Bismuth for Eradication of* Helicobacter pylori*

**DOI:** 10.1155/2018/4320219

**Published:** 2018-02-05

**Authors:** Lei Li, FanDong Meng, Shengtao Zhu, ShuiLong Guo, YongJun Wang, Xin Zhao, YiLin Sun, Yan Zhang, QinQin Wang, HuFeng Xu, ShuTian Zhang

**Affiliations:** ^1^Department of Gastroenterology, Beijing Friendship Hospital, Capital Medical University, National Clinical Research Center for Digestive Diseases, Beijing Digestive Disease Center, Beijing Key Laboratory for Precancerous Lesion of Digestive Diseases, Beijing 100050, China; ^2^Department of Digestive Diseases, Affiliated Hospital for Wei Fang Medical University, Wei Fang 261031, China; ^3^Key Laboratory of Safety Risk Assessment of Ministry of Health, China National Center for Food Safety Risk Assessment, Beijing 100021, China; ^4^Department of Ultrapathology of Beijing Neurosurgical Institute, Beijing 100050, China; ^5^Department of Ultrapathology of Beijing Neurosurgical Institute, Affiliated Bayi Brain Hospital, Army General Hospital of PLA, Beijing 100700, China; ^6^The Animal Experiment Center of Beijing Friendship Hospital, Capital Medical University, Beijing 100050, China

## Abstract

Bismuth-containing quadruple therapy has been recommended as the first line of treatment in areas of high clarithromycin or metronidazole resistance. However, safety concerns of bismuth agents have long been raised. We first assessed the efficacy and safety of Wei Bi Mei granules, which are bismuth compounds consisting of three synthetic drugs and five medicinal herbs, compared to bismuth aluminate and colloidal bismuth subcitrate (CBS) in* H. pylori*-infected mouse model. We then used atomic fluorescence spectroscopy and autometallography to measure the accumulation of three bismuth agents in the brain, heart, liver, and kidneys in adult Sprague-Dawley rats. We also evaluated the safety of bismuth agents by conducting clinical biochemistry tests in blood samples of experimental animals. Wei Bi Mei granules exhibited the highest efficacy of anti-*H. pylori* activity and yielded the lowest bismuth accumulation when compared to CBS and bismuth aluminate. Our findings show that Wei Bi Mei granules are a safe Chinese medicinal herb with potent anti-*H. pylori* activity and can be considered as an alternative to current bismuth compounds. Thus, Wei Bi Mei granules merit further evaluation, particularly with regard to efficacy and safety when they are combined with other* H. pylori* eradication medications in the clinical setting.

## 1. Introduction

*Helicobacter pylori (H. pylori)* infects at least 50% of the world's population [1] and its prevalence varies greatly depending on the studied population. The infection is more common in developing countries, with up to 80% prevalence [2]. In China, prevalence of* H. pylori* infection is as high as 60% in rural areas and is almost 50% in urban areas, although an overall decreased trend in the prevalence has been observed in recent years [3].* H. pylori* poses a significant public health problem associated with several gastroduodenal disorders, including but not limited to dyspepsia, peptic ulcer disease, and gastric malignancies [4].* H. pylori* eradication is therefore recommended for infected individuals with or without certain conditions, such as peptic ulcer disease, functional dyspepsia, and mucosa-associated lymphoid tissue type lymphoma and those with a family history of gastric cancer [5]. In particular, a population-based test-and-treat strategy for* H. pylori* is considered an effective approach for gastric cancer prevention in communities with high incidences of gastric cancer.

The recommended first-line treatment regimen for eradication of* H. pylori* infection is a triple therapy, which consists of a proton pump inhibitor (PPI) and two antibiotics, including amoxicillin or metronidazole and clarithromycin, given twice daily for at least seven days [5, 6]. However, the effectiveness of the triple* H*.* pylori* regimen has dropped to an unacceptably low level, primarily due to increasing global antibiotic resistance rates, particularly in developing countries [7]. In China, for example, the resistance to clarithromycin significantly increased from 8.6% to 20.7% and the resistance to metronidazole remained as high as 50% between 2000 and 2009 in Shanghai [6]. The overall resistance to clarithromycin and metronidazole increased annually from 14.8% to 65.4% and 38.9% to 78.8% between 2000 and 2009, respectively [8]. Bismuth, in contrast, is among one of the few antimicrobials to which resistance does not exist.* H. pylori* is highly susceptible to bismuth compounds although the mechanisms are unclear. Therefore, bismuth-containing quadruple therapy is considered an alternative therapy, particularly in regions with high clarithromycin resistance [5]. Previous meta-analyses showed that not only bismuth-containing quadruple therapy overcame clarithromycin resistance with a 78%–90% eradication rate, but also its efficacy was not affected by metronidazole resistance [9].

The most commonly used bismuth compounds are colloidal bismuth subcitrate (CBS), colloidal bismuth pectin, and bismuth aluminate. While bismuth compounds are usually considered a relatively safe drug with <1% absorption systematically, several toxicological effects ranging from erythema to nephro- and neurotoxicity have been reported [1, 10]. Given the success of bismuth-containing quadruple therapy in recent years, it is necessary to assess its efficacy and safety among different bismuth compounds. Wei Bi Mei granules, which are bismuth compounds, consist of three synthetic drugs, including bismuth aluminate, heavy magnesium carbonate, sodium hydrogen carbonate, and five medicinal herbs (250, 15, 15, 30, and 12.5 mg in one tablet, resp.). Wei Bi Mei has been used with extract licorice, cortex frangulae, fructus foeniculi, aloe, and* Acorus gramineus* to treat several gastrointestinal disorders, such as dyspepsia, gastritis, and peptic ulcer disease in China. However, data on the efficacy of* H. pylori* eradication and its side effects are scarce.

Herein, we assessed the efficacy and safety of Wei Bi Mei as an anti-*H. pylori* agent* in vivo*, comparing Wei Bi Mei to the most common bismuth compounds, bismuth aluminate, and CBS.

## 2. Experimental Design

### 2.1. The Efficacy of Bismuth Agents in a* H. pylori*-Infected Mouse Model

#### 2.1.1. Bacterial Strain and Inoculation

We utilized the mouse-adapted* H. pylori* Sydney strain 1 (strain SS1) [11] (kindly provided by Professor Chun-Jie Liu, Academy of Military Medical Sciences of the Chinese PLA, Beijing, China) in this study. Bacteria were grown under humidified microaerophilic conditions (5% O_2_, 10% CO_2_, 85% N_2_, a water jacketed CO_2_ incubator, Nuaire, UK) for 36–48 h at 37°C on Campylobacter Base Agar plates supplemented with 10% fetal calf serum (Gibco, United States), 0.38 mg L^−1^ polymyxin B, 10 mg L^−1^ vancomycin, and 2 mg L^−1^ amphotericin B. The growth status was assessed by carbol fuchsin staining. Each plate was then resuspended in normal sterile saline when bacterial suspension was harvested [11, 12].

#### 2.1.2. Animals

Specific-pathogen-free (SPF) male 10-week-old C57BL/6 mice [13] weighing between 19 and 22 g were purchased from the Vital River Laboratory Animal Technology Co. Ltd. (Charles River China, Beijing, China). Mice were housed in 23 ± 2°C standard laboratory conditions with a 12:12 h light-dark cycle. Mice were permitted free access to distilled water and sterilized standard mouse feed (Beijing Keaoxieli Feed Co. Ltd, Beijing, China) in the animal experiment center of the Chinese Center for Disease Control and Prevention. All animal experimentation was conducted in accordance with the institutional guidelines with approval of the Laboratory Animals Ethics Committee (Beijing Friendship Hospital, Capital Medical University, Beijing, China).

#### 2.1.3. Mouse Model of* H*.* pylori* Infection

Mice were randomly assigned to five groups (*n* = 6 per group); six mice were used as controls, receiving only 300 ul sterilized saline, and the remaining mice were inoculated with* H. pylori*. Mice were gavaged with 3% NaHCO_3_ to increase the stomach pH following a 12 h period of fasting, after which mice were inoculated orogastrically with 10^8^ colony forming units (CFU) of* H. pylori* in 300 ul sterilized saline for 30 min and 4 h, respectively. Blood samples from all mice were obtained and anti-*H. pylori* IgG values in sera were measured by enzyme-linked immunosorbent assays (ELISA) at 3 months after infection.

**Figure 1:**
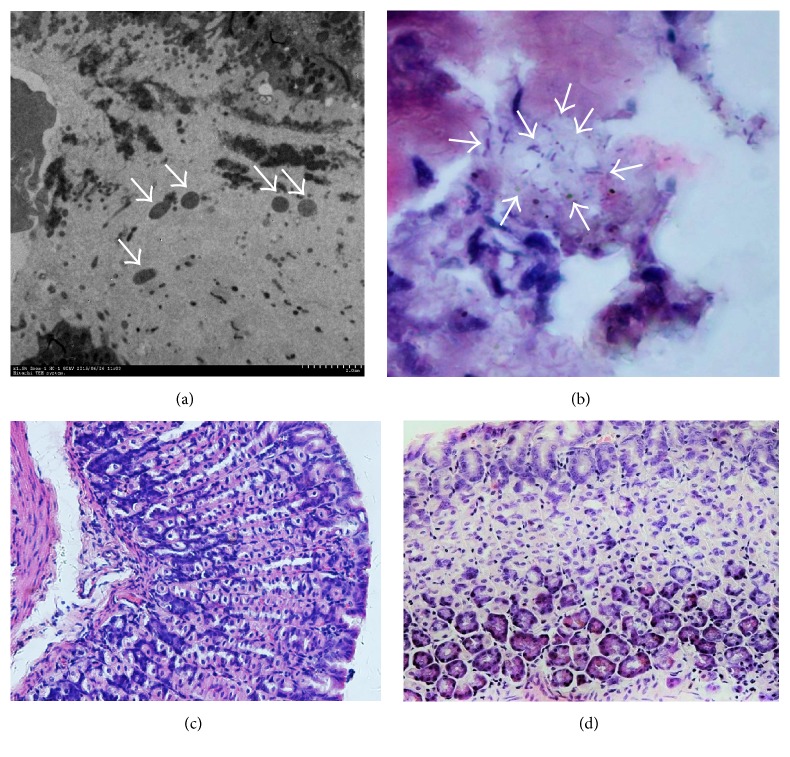
Electron microscopy (a) and H&E stain (b) show* H. pylori* colonization in the gastric mucosa of infected mice (arrows); H&E stain shows the lymphocytes and illustrates the chronic inflammation in* H. pylori*-infected mice (d) compared to uninfected mice (c).

Electron microscopy and hematoxylin and eosin (H&E) staining revealed that* H. pylori* successfully infected the gastric mucosae in mice at 3 months after infection.* H. pylori* were frequently found in the mucosa, particularly at the epithelium (Figures 1(a) and 1(b)). Whereas lymphocytes were not observed in the control group (Figure 1(c)), we observed a significant inflammatory response in the infected mice (Figure 1(d)).

*H. pylori* infected mice were further divided into four groups, with six mice in each group. The mice were given (1) distilled water (300 ul); (2) Wei Bi Mei granules (Holwray Pharmaceutical Company Limited, China), administrated at a dose of 1,454 mg/kg of body weight to achieve a dose of 131.44 mg/kg bismuth (300 ul); (3) bismuth aluminate (Holwray Pharmaceutical Company Limited, China), administrated at a dose of 248 mg/kg of body weight to achieve a dose of 131.44 mg/kg bismuth (300 ul); and (4) CBS (Lizhudele, Livzon Pharmaceutical Group Co., Ltd.), administrated at a dose of 124 mg/kg of body weight to achieve a dose of 131.44 mg/kg bismuth (300 ul). The dose of Wei Bi Mei granules, bismuth aluminate, and CBS was equivalent to the standard dose used in humans. All mice were given the drugs once in a single oral dose for 14 consecutive days. No mice died during the experimental protocol. All mice were sacrificed 14 weeks after* H. pylori* infection. The stomachs were resected into pieces, half of which were fixed in 10% formalin, and the remaining pieces were stored in liquid nitrogen.

#### 2.1.4. Histopathological Evaluation of Tissue Sections

To evaluate inflammatory cell infiltration after* H. pylori* infection, the stomach samples were fixed in 10% formalin and embedded in paraffin. Tissue sections (4 um) were stained with H&E, and* H. pylori* colonization was assessed by light microscopy. The stomach samples were cut into 1 mm pieces, placed into 4% paraformaldehyde for 2 h before rinsing three times in 0.1 M phosphate-buffered saline (PBS) for 10 min. Tissues were stored in PBS for ultrastructural studies. The tissues were cut, placed in 0.5% osmium, embedded in epon, and counter stained with uranyl acetate for electron microscopy.

### 2.2. *H. pylori *Quantification to Evaluate the Effect of Bismuth Drugs on* H. pylori *Eradication by Colony-Forming Assay

#### 2.2.1. Colony-Forming Assay

*H*.* pylori* infection levels within mouse stomach tissues were quantified using the colony-forming assay. About half of the stomachs were weighed and homogenized. One in ten serial dilutions were prepared in* Brucella* broth. Aliquots of 100 ul were spread over* H. pylori*-suitable agar plates, a campylobacter agar base with 10% fetal calf serum supplemented with 0.3 mg L^−1^ polymyxin B, 10 mg L^−1^ vancomycin, 2 mg L^−1^ amphotericin B, 5 mg L^−1^ trimethoprim, and 50 mg L^−1^ bacitracin. The number of colonies was counted after 5–7 days of incubation to determine the CFUs per gram of stomach tissue [11].

#### 2.2.2. Detection of* H. pylori* in Gastric Tissues

We extracted the DNA from mouse gastric tissues using the DNA purification kit (TIANGEN Biotech Co., Ltd., Beijing, China) following the manufacturer's instructions. The relative density of* H. pylori* was quantified as the detection of the* H. pylori*-specific ure-B DNA by semiquantitative polymerase chain reaction (PCR) using* H. pylori*-specific ure-B based primers. The primer sequences were as follows: forward primer: 5′-ACTTTATTGGCTGGTTTA-3′; reverse primer: 5′-TGGGATTAGCGAGTATGT-3′. The amount of mouse glyceraldehyde-3-phosphate dehydrogenase (GAPDH) DNA in the same specimen was measured for normalization. The primers for detection of mouse GAPDH DNA were as follows: forward primer: 5′-AATGGATTTGGACGCATTGGT-3′; reverse primer: 5′-TTTGCACTGGTACGTGTTGAT-3′. The relative density of* H. pylori* in the samples was expressed as the ratio of* H. pylori*-specific ure-B to GAPDH DNA.

### 2.3. The Accumulation of Bismuth Agents in Vital Organs

#### 2.3.1. Animals

Ninety-six SPF adult male Sprague-Dawley rats [14], weighing between 250 and 300 g were purchased from the Vital River Laboratory Animal Technology Co. Ltd. (Charles River China, Beijing, China). All rats were housed in 23 ± 2°C, standard laboratory conditions with a 12:12 h light-dark cycle, and were given sterilized standard rat feed and distilled water. The animals were housed in the animal experiment center of Beijing Friendship Hospital, Capital Medical University, Beijing, China. All rats were weighed once a week and observed daily for signs of appetite and neurological changes, that is, ataxic gait, tremor, or coordination disorders.

#### 2.3.2. Administration of Bismuth Agents

All rats were randomly assigned to four groups (*n* = 24 per group). The rats in the control group (Group A) were inoculated intragastrically with distilled water only. The remaining groups (Groups B, C, and D) were given three bismuth agents: Group B received Wei Bi Mei granules at a dose of 1,454 mg/kg of body weight; group C received bismuth aluminate at a dose of 248 mg/kg of body weight; and group D received CBS at a dose of 124 mg/kg of body weight. The maximum dose, which was equivalent to 2 times the dose used in humans, was applied for Wei Bi Mei granules and bismuth aluminate to evaluate the drug accumulation in vital organs and the recovery after drug withdrawal. The dose of CBS was equivalent to the standard dose used in humans. All animals were given the drugs once in a single dose for 8 weeks. No rats died during the 8-week period. We did not observe significant changes in food intake and weight between groups at any time point. None of the bismuth-exposed rats showed any signs of altered neurological behavior, such as tremor, coordination disorders, ataxic gait, or hindlimb incoordination [15].

#### 2.3.3. Sample Preparation

All rats were deeply anaesthetized with 50 mg/kg pentobarbital and sacrificed by transcardial perfusion using 4% paraformaldehyde in PBS at 0, 4, 8, and 12 weeks after bismuth compound exposure for 8 weeks. Blood samples (2 ml) from intracardiac puncture and brain, heart, liver, and kidneys were collected at each time point. Serum, obtained from blood samples by centrifugation for 10 min, was used to test liver function, renal function, myocardial enzymes, and electrolytes. Tissue samples of brain, heart, liver, and kidneys were fixed in 10% formalin, homogenized, and weighed for quantitative bismuth measurements, and the samples were then stored at −20°C for hydride generation atomic fluorescence spectrometry (HG-AFS) and autometallographically (AMG).

#### 2.3.4. Hydride Generation Atomic Fluorescence Spectrometry (HG-AFS)

The AFS-9800 atomic fluorescence spectrometry (Beijing Haiguang Instrument Co., Beijing, China) was used in this study to quantify bismuth accumulation. A series of standard solutions (0.0, 1.0, 2.0, 4.0, 8.0, and 10.0 ug/L) were prepared just before use by diluting the bismuth stock solution (100 mg/L). We used microwave-assisted digestion to decompose samples. A total of 2.0 g of homogenized samples were added to digestion vessels containing 10 mL nitric acid and 1 ml perchloric acid, which were then put into the microwave oven for digestion. The heating program was 105°C for 10 min, 140°C for 10 min, and 190°C for 10 min. The final digests were diluted with purified water. The operation system of the HG-AFS is shown in Table 1.

#### 2.3.5. Autometallography (AMG)

We used AMG since it is a highly sensitive method for tracing bismuth agents in tissues [16]. The tissue blocks of brain, heart, liver, and kidneys were embedded in paraffin and cut into 4 um sections and placed on special adhesion microscope glass slides for AMG development. The AMG developer included 60 ml 50% gum Arabic colloid, 10 ml citrate buffer (pH 3.7), 15 ml hydroquinone solution, 15 ml silver ion, and 100 ml 5% sodium thiosulfate solution. We then carefully mixed 60 ml gum Arabic colloid, 10 ml citrate buffer, and 15 ml of hydroquinone (avoiding exposure to light) in a 100 ml cleaned vessel adding 15 ml silver ion immediately before use. The sample sections were developed at 26°C for at least 60 min. AMG development was terminated by replacing the developer with a 5% sodium thiosulfate solution for 10 min. The vessel was then placed under running 40°C water for 20 min, rinsed twice in distilled water, and stained with hematoxylin.

We used the Shapiro-Wilk test to assess the normality of the data. As the data were normally distributed (data not shown), we described the data using mean ± standard deviation (SD). Bismuth accumulation in the tissues among different groups was compared using ANOVA. The parameters found to be significant in ANOVA were assessed by the Dunnett test. Serum biochemical values were compared using independent *t*-test among different groups. A two-tailed *P* value < 0.05 was considered statistically significant. Data were analyzed using SPSS 20.0 (IBM Corporation, Somers, NY).

## 3. Results

We did not observe significant changes in food intake and weight changes in any of the rat groups. None of the bismuth-exposed rats showed any signs of altered neurological behaviors, such as tremor, coordination disorders, ataxic gait, or hindlimb incoordination [15].

### 3.1. Eradication Efficacy of Three Bismuth Agents in* H. pylori*-Infected Mice

**Figure 2:**
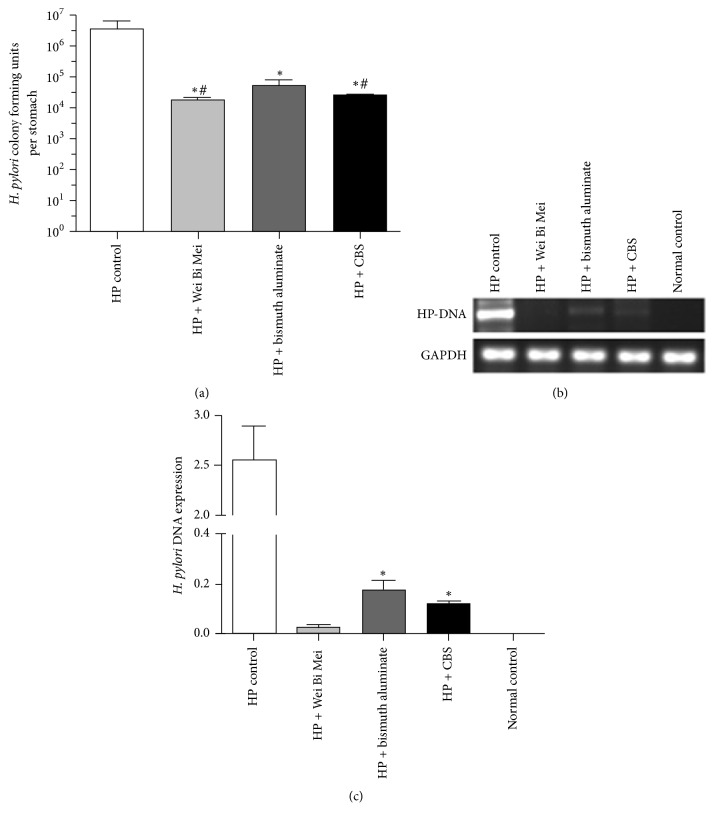
The effect of three bismuth drugs on* H. pylori* eradication. (a) Colony-forming assay shows that* H. pylori* colonization levels were decreased in the three bismuth drug groups, especially in the Wei Bi Mei granules and CBS groups. ^*∗*^*P* < 0.05 versus HP control, ^#^*P* < 0.01 versus HP + bismuth aluminate. (b) and (c)* H. pylori*-specific DNA gene expression was significantly lower in the Wei Bi Mei granules group compared to the CBS and bismuth aluminate groups. The* H. pylori* DNA gene expression levels were all lower in the three bismuth groups compared to the control group without any bismuth treatment. ^*∗*^*P* < 0.01 versus HP + Wei Bi Mei.

Our colony-forming assay showed that the number of* H. pylori*, quantitated by culture and expressed as log^10^ CFUs per gram of stomach, decreased in all three bismuth drug groups compared to the control group (Figure 2, *P* < 0.05). The number of* H. pylori* in the Wei Bi Mei granules and CBS groups was significantly lower than the bismuth aluminate group (*P* < 0.01) (Figure 2(a)), and there was no statistical difference between the Wei Bi Mei granules and CBS groups (Figure 2(a)).

Figure 2(b) shows the amplification products of* H. pylori*-specific DNA and GAPDH in three experimental groups and the control group. The* H. pylori* DNA expression level is denoted by the ratio of gray values of DNA bands with Quantity One software between* H. pylori *ure-B DNA and GAPDH (Figure 2(c)). The DNA expression level of* H. pylori *was significantly lower in the Wei Bi Mei granules group compared to the CBS and bismuth aluminate groups (*P* < 0.01), and the number* of H. pylori *was comparable between the CBS and bismuth aluminate groups (*P* = 0.15).

### 3.2. Accumulation and Evaluation of Bismuth Agents in Vital Organs

The biochemical parameters at different time points in the treatment groups and controls are shown in Tables 2–4. All parameters remained within physiological ranges at all time points after drug withdrawal. No significant differences were observed among all bismuth-treated groups and controls with regard to blood chemistry, including alanine aminotransferase activity, alkaline phosphatase activity, total protein, albumin, total bilirubin, direct bilirubin, glucose, creatinine, and blood urea nitrogen. There were some significant variations in several parameters (aspartate aminotransferase activity, potassium ions, sodium ions, and chloride ions); however, all of these parameters remained within physiological ranges.

No bismuth was detected in any tissue samples in the control group. After 8 weeks of exposure, bismuth deposits were mainly found in the kidneys, followed by liver, brain, and heart in all treatment groups. Wei Bi Mei granules yielded the lowest accumulation of bismuth in the kidneys, brain, heart, and liver compared to CBS and bismuth aluminate. Bismuth accumulation gradually decreased in all tissues over time. Bismuth was still found in all tissues, mainly in the kidneys in the CBS group, but it was hardly detected in the Wei Bi Mei granules group after 8 weeks after drug withdrawal. After 12 weeks after drug withdrawal, bismuth was not detected in the tissues of the Wei Bi Mei granules group, but it was detected in all tissues, particularly in the kidneys, in the CBS group (Table 5). After 8 weeks of bismuth exposure, bismuth accumulation in the kidneys was lower in the Wei Bi Mei group compared to the CBS and bismuth aluminate groups (*P* < 0.001).

**Figure 3:**
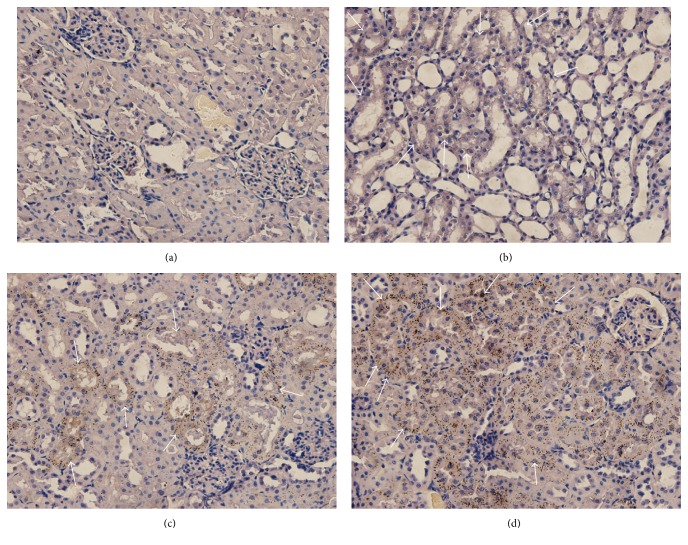
Bismuth accumulation in kidneys with the autometallography is shown by light microscopy. In panels (b), (c), and (d), the white arrows indicate the Bismuth accumulation in the kidney compared to the control group (a); bismuth accumulation in the CBS group (d) was higher compared to the Wei Bi Mei (b) and bismuth aluminate (c) groups, respectively. The accumulation of bismuth was the lowest in the Wei Bi Mei group (b).

AMG and light microscopy showed that bismuth explicitly accumulated in the kidneys in the CBS, bismuth aluminate, and Wei Bi Mei groups. No bismuth was found in the control group. Bismuth accumulated more in the CBS group than in the bismuth aluminate and Wei Bi Mei groups (Figure 3).

## 4. Discussion

In this study, we demonstrated that Wei Bi Mei granules, which are bismuth compounds, have potent anti-*H. pylori* activity with relatively low accumulation of residual bismuth in vital organs, specifically in the kidneys, compared to CBS and bismuth aluminate in a mouse model. Wei Bi Mei granules have been used to treat several gastrointestinal disorders [17, 18]; however, to the best of our knowledge, this is the first study to evaluate the efficacy and safety of Wei Bi Mei granules in* H. pylori* eradication therapy.

It has long been established that bismuth has potent anti-*H. pylori* activity [19], and there is no* in vitro* resistance [20]. Therefore, bismuth has been included in double, triple, and quadruple regimens, which have increased the efficacy of* H. pylori* eradication therapy [5]. However, the underlying mechanisms of bismuth's anti-*H. pylori* effects are unclear. A competitive transport pathway involving bismuth and ferric ion has been demonstrated in* H. pylori* single cells [21]. It has been postulated that while bismuth does not directly interact with urease or the urea channel, the efficacy of growth-dependent antibiotics is enhanced as a result of increased metabolic activity of a neutralophile due to decreased cytoplasmic pH [22]. We acknowledge that the anti-*H. pylori* activity of Wei Bi Mei is mainly due to the bismuth aluminate in the Wei Bi Mei granules. However, Wei Bi Mei granules exhibited more potent anti-*H. pylori* activity than bismuth aluminate alone, indicating that the efficacy of Wei Bi Mei against* H. pylori* may be augmented by the other ingredients in the compounds. In addition to the anti-*H. pylori* activity of bismuth aluminate, the other two synthetic drugs, including heavy magnesium carbonate and sodium hydrogen carbonate, protect the gastric mucosa by increasing gastric pH [23, 24]. The extract powder of licorice can relieve gastrointestinal smooth muscle spasms, and triterpenes and flavonoids of licorice are the main constituent of ulcer treatment. Thus, licorice extract can prevent damage of gastric mucosal cells by increasing hexose in these cells, which therefore inhibits the growth of* H. pylori in vitro* [25]. Acorus gramineus can initiate digestion and inhibit urease activity that cause stomach ache and suppress bacteria [26]. Fructus foeniculi can reduce flatulence, relieving pain, resisting inflammation, protecting cells, and regulating gastrointestinal motility in gastrointestinal disorders associated with* H. pylori* [27]. Aloes have been used to treat gastrointestinal disorders probably, likely because of their therapeutic effects that alleviate inflammation, inhibit gastric acid and pepsin secretion, inhibit gastric smooth muscle, and promote ulcer healing [28]. Therefore, we postulate that the increased efficacy of Wei Bi Mei is possibly due to the combination of the ingredients in the Wei Bi Mei granules.

Concerns regarding the safety of bismuth compounds have long been raised. Studies have shown that encephalopathy and nephrotoxicity are side effects of chronic exposure to high levels of bismuth salts and that the liver and kidneys are target organs of bismuth poisoning [29].

In this study, we showed that all parameters of blood chemistry remained within physiological ranges after exposure to all three bismuth compounds following 8 weeks of treatment. Although we did not attempt to quantify the amount of absorbed bismuth, bismuth was readily absorbed and retained mainly in the kidneys, followed by the liver, brain, and heart for all three bismuth compounds. This effect was observed after 8 weeks of exposure and gradually disappeared 12 weeks after the exposure, which is in accordance with the literature [10, 30]. Compared to bismuth aluminate and CBS, Wei Bi Mei resulted in the least accumulation of bismuth in all organs tested in our study, with a shortened time of elimination of bismuth in the kidneys. The three bismuth compounds exhibited different bismuth accumulation, possibly due to their differences in absorption. The amounts of both Wei Bi Mei granules and bismuth aluminate used in our study were 131.44 mg/kg bismuth, about twice as much as the dose used in humans, whereas the amounts of CBS equalled 45.47 mg/kg bismuth, which is the standard dose used in humans. We observed the highest bismuth accumulation in the kidneys of the CBS group compared to its counterparts. Studies have shown that the intestinal absorption of CBS was significantly higher compared to bismuth aluminate [31], which partly explains the high accumulation of bismuth in the kidneys of the CBS group compared to the other two agents in the present study. Interestingly, the accumulation of bismuth was significantly lower in Wei Bi Mei granules compared to bismuth aluminate, although both agents consisted of the same amount of bismuth. Studies have shown that Acorus gramineus has an excitatory effect on isolated gastric antrum and pyloric annular muscles, which results in enhanced intestinal peristalsis [32]. Fructus foeniculi can reduce gastric tension, promote peristalsis normalization, and relieve pain [32]. Cortex frangulae and aloe can promote intestinal peristalsis and purgation [32, 33]. This is possibly due to the fact that medicinal herbs in Wei Bi Mei may have underlying mechanisms that lead to less absorption and less accumulation in vital organs.

## 5. Conclusion

In this study, we examined and compared the efficacy and safety among three different bismuth compounds. We showed that Wei Bi Mei exhibited the highest anti-*H. pylori* efficacy and yielded the lowest accumulation of bismuth in the kidneys compared to CBS and bismuth aluminate. Wei Bi Mei, which consists of bismuth compounds, merits further evaluation for its clinical implications.

## Figures and Tables

**Table 1:** Parameters of atomic fluorescence spectrometry.

Parameters	Setting value
Bismuth hollow cathode lamp	80 Ma
Negative voltage of PMT	300 V
Observation height	8 mm
Flow rate of carrier gas	0.4 L min^−1^
Flow rate of shield gas	1.0 L min^−1^
Read mode	Peak area
Measurement method	Standard curve

**Table 2:** Serum biochemical values in rats orally treated with Wei Bi Mei at withdrawal at 0, 4, 8, and 12 weeks after bismuth exposure.

Parameter	Time							
	0		4 w		8 w		12 w	
	Wei Bi Mei	Water	Wei Bi Mei	Water	Wei Bi Mei	Water	Wei Bi Mei	Water
ALT (U/L)	40 (6)	38 (3)	42 (4)	47 (6)	43 (5)	41 (6)	41 (3)	42 (3)
AST (U/L)	100 (27)	134 (23)	110 (39)^*∗*^	116 (29)	97 (31)	82 (10)	112 (25)	109 (21)
ALP (U/L)	163 (24)	183 (67)	206 (57)	207 (50)	150 (29)	153 (31)	155 (30)	172 (77)
GLU (mmol/L)	5.8 (0.9)	5.2 (0.4)	5.45 (0.8)	5.6 (0.5)	5.85 (0.8)	5.2 (0.3)	5.2 (0.4)	5.4 (0.4)
TP (g/L)	56 (6)	56 (3)	55 (4)	55 (3)	53 (3)	57 (4)	54 (5)	54 (3)
ALB (g/L)	28 (2)	29 (20)	29 (2)	28 (3)	28 (1)	27 (2)	28 (1)	30 (2)
T-BIL (*µ*mol/L)	7.35 (1.89)	7.26 (1.96)	7.48 (1.44)	6.68 (1.25)	6.58 (1.78)	7.95 (1.01)	7.21 (1.13)	7.47 (1.38)
D-BIL (*µ*mol/L)	4.01 (0.94)	4.6 (1.32)	3.98 (1.37)	4.06 (0.72)	3.75 (1.16)	5.28 (0.49)	3.93 (0.91)	4.08 (0.96)
Cre (*µ*mol/L)	38 (5)	40 (3)	36 (5)	33 (8)	30 (6)	33 (6)	36 (5)	37 (4)
Bun (mmol/L)	5.70 (1.25)	6.00 (1.26)	6.03 (0.83)	6.57 (0.98)	6.21 (1.65)	7.28 (2.47)	5.91 (0.667)	6.38 (1.16)
Na^+^ (mmol/L)	141.2 (4.7)	144.3 (2.0)	143.4 (4.1)	143.9 (1.73)	141.4 (2.9)	141.7 (2.5)	140 (4.0)	139 (1.4)
K^+^ (mmol/L)	4.23 (0.39)	4.31 (0.43)	4.62 (0.49)	4.59 (0.47)	4.58 (0.48)	4.30 (0.34)	4.11 (0.31)	4.56 (0.33)
Cl^−^ (mmol/L)	100.2 (4.2)	97.7 (2.1)	103.5 (5.2)^*∗∗*^	96.5 (1.9)	100.7 (4.7)	96.8 (1.5)	99.6 (4.7)	100.4 (1.7)

Alanine aminotransferase activity (ALT), aspartate aminotransferase activity (AST), alkaline phosphatase activity (ALP), glucose (GLU), total protein (TP), albumin (ALB), total bilirubin (T-BIL), direct bilirubin (D-BIL), creatinine (CRE), blood urea nitrogen (BUN), potassium ions (K^+^), sodium ions (Na^+^), and chloride ions (Cl^−^). Colloidal bismuth subcitrate (CBS); values are mean (SD); ^*∗*^*P* < 0.05 and ^*∗∗*^*P* < 0.01 when compared to the controls using the independent *t*-test.

**Table 3:** Serum biochemical values in rats orally treated with bismuth aluminate at withdrawal at 0, 4, 8, and 12  weeks after bismuth exposure.

Parameter	Time							
	0		4 w		8 w		12 w	
	Bismuth aluminate	Water	Bismuth aluminate	Water	Bismuth aluminate	Water	Bismuth aluminate	Water
ALT (U/L)	40 (3)	38 (3)	45 (3)	47 (6)	38 (5)	41 (6)	39 (3)	42 (3)
AST (U/L)	128 (24)	134 (23)	94 (35)	116 (29)	95 (14)	82 (10)	97 (23)	109 (21)
ALP (U/L)	139 (51)	183 (67)	174 (79)	207 (50)	169 (59)	153 (31)	172 (77)	172 (77)
GLU(mmol/L)	5.31 (0.9)	5.2 (0.4)	5.76 (5.55)	5.6 (0.5)	5.55 (0.81)	5.2 (0.3)	5.4 (0.5)	5.4 (0.4)
TP (g/L)	53 (3)	56 (3)	55 (4)	55 (3)	56 (3)	57 (4)	54 (3)	54 (3)
ALB (g/L)	27 (2)	29 (2)	29 (2)	28 (3)	28 (2)	27 (2)	30 (1)	30 (2)
T-BIL(*µ*mol/L)	7.03 (2.01)	7.26 (1.96)	7.2 (1.59)	6.68 (1.25)	7.08 (1.47)	7.95 (1.01)	7.30 (1.21)	7.47 (1.38)
D-BIL(*µ*mol/L)	4.20 (1.09)	4.60 (1.32)	4.70 (1.27)	4.06 (0.72)	4.08 (1.05)	5.28 (0.49)	4.02 (0.73)	4.08 (0.96)
Cre (*µ*mol/L)	41 (4)	40 (3)	31 (5)	33 (8)	32 (10)	33 (6)	36 (4)	37 (4)
Bun (mmol/L)	5.63 (0.70)	6.00 (1.26)	7.51 (0.71)	6.57 (0.98)	6.09 (1.03)	7.28 (2.47)	5.85 (0.99)	6.38 (1.16)
Na^+^ (mmol/L)	143 (1.8)	144 (2.0)	140 (1.2)^*∗∗*^	143.9 (1.73)	142.7 (2.9)	142 (2.5)	143 (2.4)	139 (1.4)
K^+^ (mmol/L)	5.00 (0.25)^*∗∗*^	4.31 (0.43)	4.67 (0.35)	4.59 (0.47)	4.02 (0.33)	4.30 (0.34)	4.47 (0.35)	4.56 (0.33)
Cl^−^ (mmol/L)	102 (5.1)	97.7 (2.1)	101 (4.6)^*∗*^	96.5 (1.9)	96.9 (3.4)	96.8 (1.5)	98.3 (3.2)	100 (1.7)

Alanine aminotransferase activity (ALT), aspartate aminotransferase activity (AST), alkaline phosphatase activity (ALP), glucose (GLU), total protein (TP), albumin (ALB), total bilirubin (T-BIL), direct bilirubin (D-BIL), creatinine (CRE), blood urea nitrogen (BUN), potassium ions (K^+^), sodium ions (Na^+^), and chloride ions (Cl^−^). Colloidal bismuth subcitrate (CBS); values are mean (SD); ^*∗*^*P* < 0.05 and ^*∗∗*^*P* < 0.01 when compared to the controls using the independent *t*-test.

**Table 4:** Serum biochemical values in rats orally treated with CBS at withdrawal at 0, 4, 8, and 12 weeks after bismuth exposure.

Parameter	Time							
	0		4 w		8 w		12 w	
	CBS	Water	CBS	Water	CBS	Water	CBS	Water
ALT (U/L)	40 (8)	38 (3)	46 (6)	47 (6)	39 (4)	41 (6)	42 (6)	42 (3)
AST (U/L)	84 (13)^*∗∗*^	134 (23)	131 (40)	116 (29)	98 (25)	82 (10)	123 (22)	109 (21)
ALP (U/L)	187 (54)	183 (67)	202 (31)	207 (50)	209 (47)	153 (31)	151 (29)	172 (77)
GLU (mmol/L)	5.4 (0.4)	5.2 (0.4)	5.90 (0.8)	5.6 (0.5)	5.5 (0.6)	5.2 (0.3)	5.1 (0.4)	5.4 (0.4)
TP (g/L)	53 (3)	56 (3)	54 (2)	55 (3)	53 (3)	57 (4)	54 (2)	54 (3)
ALB (g/L)	30 (1)	29 (2)	27 (2)	28 (3)	28 (3)	27 (2)	27 (2)	30 (2)
T-BIL (*µ*mol/L)	8.36 (0.93)	7.26 (1.96)	8.13 (1.06)	6.68 (1.25)	7.58 (1.60)	7.95 (1.01)	7.4 (1.29)	7.47 (1.38)
D-BIL (*µ*mol/L)	5.36 (0.93)	4.6 (1.32)	4.96 (1.07)	4.06 (0.72)	4.05 (1.06)	5.28 (0.49)	4.02 (0.64)	4.08 (0.96)
Cre (*µ*mol/L)	38 (7)	40 (3)	34 (4)	33 (8)	30 (5)	33 (6)	36 (4)	37 (4)
Bun (mmol/L)	5.56 (1.01)	6.00 (1.26)	6.96 (1.42)	6.57 (0.98)	6.43 (0.97)	7.28 (2.47)	5.80 (0.51)	6.38 (1.16)
Na^+^ (mmol/L)	143 (2.3)	144 (2.0)	140 (1.50)^*∗∗*^	144 (1.73)	142 (2.3)	142 (2.5)	142 (2.3)	139 (1.4)
K^+^ (mmol/L)	4.43 (0.56)	4.31 (0.43)	4.37 (0.70)	4.59 (0.47)	4.75 (0.33)	4.30 (0.34)	4.21 (0.45)	4.56 (0.33)
Cl^−^ (mmol/L)	98.4 (0.8)	97.7 (2.1)	98.1 (2.70)	96.5 (1.9)	99.2 (1.9)	96.8 (1.5)	101 (2.0)	100 (1.7)

Alanine aminotransferase activity (ALT), aspartate aminotransferase activity (AST), alkaline phosphatase activity (ALP), glucose (GLU), total protein (TP), albumin (ALB), total bilirubin (T-BIL), direct bilirubin (D-BIL), creatinine (CRE), blood urea nitrogen (BUN), potassium ions (K^+^), sodium ions (Na^+^), chloride ions (Cl^−^). Colloidal bismuth subcitrate (CBS); values are mean (SD); ^*∗*^*P* < 0.05  and  ^*∗∗*^*P* < 0.01 when compared to the controls using the independent *t*-test.

**Table 5:** Bismuth accumulation in kidney, heart, liver, and brain.

	Wei Bi Mei	Bismuth aluminate	CBS	*P* ^2^
*Kidney*				
Lavage 8 w	785.9 (14.6)	3268.0 (205)	18205 (506)	<0.001
Lavage 8 w withdraw 4 w	192.9 (6.67)	269.5 (14.8)	3399 (189)	<0.001
Lavage 8 w withdraw 8 w	37.7 (4.97)	63.4 (5.67)	315 (28.5)	<0.001
Lavage 8 w withdraw 12 w	3.73 (1.51)	14.4 (1.81)	91.4 (6.63)	<0.001
*Heart*				
Lavage 8 w	5.43 (1.82)	52.4 (2.53)	73.6 (4.80)	<0.001
Lavage 8 w withdraw 4 w	2.19 (0.89)	8.32 (1.14)	28.2 (4.93)	<0.001
Lavage 8 w withdraw 8 w	1.93 (0.94)	6.03 (1.44)	10.0 (2.42)	<0.001
Lavage 8 w withdraw 12 w	0.98 (0.40)	1.04 (0.45)	1.00 (0.69)	0.552
*Liver*				
Lavage 8 w	16.8 (2.22)	102.8 (3.05)	398 (5.70)	<0.001
Lavage 8 w withdraw 4 w	2.22 (1.16)	7.65 (2.11)	66.0 (2.39)	<0.001
Lavage 8 w withdraw 8 w	4.55 (1.39)	4.97 (1.20)	44.5 (6.60)	<0.001
Lavage 8 w withdraw 12 w	1.29 (0.63)	1.47 (0.54)	7.05 (2.56)	<0.001
*Brain*				
Lavage 8 w	5.14 (0.81)	95.6 (1.99)	120.3 (4.97)	<0.001
Lavage 8 w withdraw 4 w	4.69 (2.50)	10.2 (2.94)	17.2 (3.65)	<0.001
Lavage 8 w withdraw 8 w	4.55 (1.39)	4.97 (1.20)	14.3 (1.58)	<0.001
Lavage 8 w withdraw 12 w	1.29 (0.65)	4.46 (2.13)	5.36 (2.26)	0.004

CBS: colloidal bismuth subcitrate; values are mean (SD). ^2^One-way ANOVA was used to compare the values of three groups.

## References

[1] Leja M., Axon A., Brenner H. (2016). Epidemiology of Helicobacter pylori infection. *Helicobacter*.

[2] Ford A. C., Axon A. T. R. (2010). Epidemiology of Helicobacter pylori infection and Public Health Implications. *Helicobacter*.

[3] Nagy P., Johansson S., Molloy-Bland M. (2016). Systematic review of time trends in the prevalence of Helicobacter pylori infection in China and the USA. *Gut Pathogens*.

[4] Hunt R. H., Xiao S. D., Megraud F. (2010). *World Gastroenterology Organisation Global Guidelines, Helicobacter pylori in Developing Countries*.

[5] Malfertheiner P., Megraud F., O'Morain C. A. (2017). Management of Helicobacter pylori infection-the Maastricht V/Florence Consensus Report. *Gut*.

[6] Sun Q.-J., Liang X., Zheng Q. (2010). Resistance of *Helicobacter pylori* to antibiotics from 2000 to 2009 in Shanghai. *World Journal of Gastroenterology*.

[7] Graham D. Y., Fagoonee S., Pellicano R. (2017). Increasing role for modified bismuth-containing quadruple therapies for Helicobacter pylori eradication. *Minerva Gastroenterologica e Dietologica*.

[8] Gao W., Cheng H., Hu F. (2010). The evolution of Helicobacter pylori antibiotics resistance over 10 years in Beijing, China. *Helicobacter*.

[9] Venerito M., Krieger T., Ecker T., Leandro G., Malfertheiner P. (2013). Meta-analysis of bismuth quadruple therapy versus clarithromycin triple therapy for empiric primary treatment of Helicobacter pylori infection. *Digestion*.

[10] Slikkerveer A., de Wolff F. A. (1989). Pharmacokinetics and toxicity of bismuth compounds. *Medical Toxicology and Adverse Drug Experience*.

[11] Wan X.-K., Yuan S.-L., Tao H.-X. (2016). The Upregulation of TRAF1 Induced by Helicobacter pylori Plays an Antiapoptotic Effect on the Infected Cells. *Helicobacter*.

[12] Lee A., O'Rourke J., De Ungria M. C., Robertson B., Daskalopoulos G., Dixon M. F. (1997). A standardized mouse model of Helicobacter pylori infection: Introducing the Sydney strain. *Gastroenterology*.

[13] Correia M., Michel V., Matos A. A. (2012). Docosahexaenoic acid inhibits *Helicobacter pylori* growth in vitro and mice gastric mucosa colonization. *PLoS ONE*.

[14] Swy E. R., Schwartz-Duval A. S., Shuboni D. D. (2014). Dual-modality, fluorescent, PLGA encapsulated bismuth nanoparticles for molecular and cellular fluorescence imaging and computed tomography. *Nanoscale*.

[15] Larsen A., Stoltenberg M., Søndergaard C., Bruhn M., Danscher G. (2005). In vivo distribution of bismuth in the mouse brain: Influence of long-term survival and intracranial placement on the uptake and transport of bismuth in neuronal tissue. *Basic & Clinical Pharmacology & Toxicology*.

[16] Danscher G., Stoltenberg M., Kemp K., Pamphlett R. (2000). Bismuth autometallography: Protocol, specificity, and differentiation. *Journal of Histochemistry & Cytochemistry*.

[17] Chen J. Y., Hen J. Y., Huang P. N. (2017). Effect of compound Bismuth Magnesium quadruple therapy in the treatment of Hp-related peptic ulcer. *China Modern Medicine*.

[18] Qiao W., Di J., Wang X. W. (2016). Effects of Chinese medicine portions of Weibimei on cell cycle regulation and tumor inhibition in nude mice. *Chinese Journal of Biochemical Pharmaceutics*.

[19] Marshall B. J., Armstrong J. A., Francis G. J., Nokes N. T., Wee S. H. (1987). Antibacterial action of bismuth in relation to Campylobacter pyloridis colonization and gastritis. *Digestion*.

[20] Midolo P. D., Lambert J. R., Kerr T. G., Tee W. (1999). In Vitro Synergy Between Ranitidine Bismuth Citrate and Tetracycline or Clarithromycin Against Resistant Strains of Helicobacter pylori. *European Journal of Clinical Microbiology & Infectious Diseases*.

[21] Tsang C.-N., Ho K.-S., Sun H., Chan W.-T. (2011). Tracking bismuth antiulcer drug uptake in single helicobacter pylori cells. *Journal of the American Chemical Society*.

[22] Marcus E. A., Sachs G., Scott D. R. (2015). Colloidal bismuth subcitrate impedes proton entry into Helicobacter pylori and increases the efficacy of growth-dependent antibiotics. *Alimentary Pharmacology & Therapeutics*.

[23] Schaefer D., Wheeler L., Noller C., Keyser R., White J. (1982). Neutralization of Acid in the Rumen by Magnesium Oxide and Magnesium Carbonate. *Journal of Dairy Science*.

[24] Bowen B. K., Krause W. J., Ivey K. J. (1977). Effect of sodium bicarbonate on aspirin-induced damage and potential difference changes in human gastric mucosa. *British Medical Journal*.

[25] Graham D. Y., Fischbach L. (2010). *Helicobacter pylori* treatment in the era of increasing antibiotic resistance. *Gut*.

[26] Lee S. Y., Moon E., Kim S. Y., Choi S. U., Lee K. R. (2013). Quinone derivatives from the rhizomes of Acorus gramineus and their biological activities. *Bioscience, Biotechnology, and Biochemistry*.

[27] Zaidi S. F., Muhammad J. S., Shahryar S. (2012). Anti-inflammatory and cytoprotective effects of selected Pakistani medicinal plants in Helicobacter pylori-infected gastric epithelial cells. *Journal of Ethnopharmacology*.

[28] Prabjone R., Thong-Ngam D., Wisedopas N., Chatsuwan T., Patumraj S. (2006). Anti-inflammatory effects of Aloe vera on leukocyte-endothelium interaction in the gastric microcirculation of Helicobacter pylori-infected rats. *Clinical Hemorheology and Microcirculation*.

[29] Karahan S., Erden A., Bulut K. (2013). A case of bismuth intoxication with irreversible renal damage. *International Journal of Nephrology and Renovascular Disease*.

[30] Tillman L. A., Drake F. M., Dixon J. S., Wood J. R. (1996). safety of bismuth in the treatment of gastrointestinal diseases. *Alimentary Pharmacology & Therapeutics*.

[31] Dresow B., Nielsen P., Fischer R., Wendel J., Gabbe E. E., Heinrich H. C. (1991). Bioavailability of bismuth from205Bi-labelled pharmaceutical oral Bi-preparations in rats. *Archives of Toxicology*.

[32] Wang J. G., Zheng M. Y. (2015). Effect of Compound Bismush and Magnesium Granules Combined with Triple Therapy for Treament of Hp Positive Duodenal Bulbar Ulcer. *China Medical Herald*.

[33] Liu Y. J., Qin M. (2015). Observation on The Efficacy of Compound bismuth and Mahnesium Granules Combined with Itopride on Chronic Gastritus with Dyspepsia Symptoms. *China Foreign Medical Treatment*.

